# Complete Genome Sequences of Four Isolates of *Plutella xylostella* Granulovirus

**DOI:** 10.1128/genomeA.00633-16

**Published:** 2016-06-30

**Authors:** Robert J. Spence, Christopher Noune, Caroline Hauxwell

**Affiliations:** Queensland University of Technology (QUT), Brisbane, Australia

## Abstract

Granuloviruses are widespread pathogens of *Plutella xylostella* L. (diamondback moth) and potential biopesticides for control of this global insect pest. We report the complete genomes of four *Plutella xylostella* granulovirus isolates from China, Malaysia, and Taiwan exhibiting pairs of noncoding, homologous repeat regions with significant sequence variation but equivalent length.

## GENOME ANNOUNCEMENT

*Plutella xylostella* L. (diamondback moth) is a globally important insect pest of *Brassica* crops that is widely resistant to insecticides incurring control costs up to five billion U.S. dollars annually (1–3). *Plutella xylostella* granuloviruses (*Plxy*GV) have potential use as biopesticides to manage insecticide resistance and improve pest management (2, 4, 5). Here, we present complete genome sequences of four isolates of *Plxy*GV from China, Malaysia, and two from Taiwan (6, 7).

Isolates were passaged through *P. xylostella* larvae and occlusion bodies purified by centrifugation, followed by incubations in 1 M sodium carbonate (Na_2_CO_3_) at room temperature for 5 min and 1% N-lauryl sarcosine at 37°C for 15 min. DNA was then purified using an Isolate II Genomic DNA kit (catalogue no. BIO-52067, Bioline) from step 4 according to manufacturer’s instructions.

Genomic DNA was prepared for sequencing using the Nextera XT kit and medium-output flow cell on an Illumina Next-Seq 500 with 150 bp paired-end sequencing. Raw sequence data comprised 12,852,411 reads (*Plxy*GV-C, China); 8,878,618 reads (*Plxy*GV-T, Taiwan); 12,251,888 reads (*Plxy*GV-K, Taiwan); and 16,077,717 reads (*Plxy*GV-M, Malaysia), representing average genome coverage of 19,088×, 13,186×, 18,196×, and 23,878×, respectively.

Read inspection, trimming, and genome assembly used the method of Noune and Hauxwell (8). Reads were analyzed and trimmed using FastQC version 0.11.3 (http://www.bioinformatics.babraham.ac.uk/projects/fastqc/) and FastX trimmer version 0.0.13 (http://hannonlab.cshl.edu/fastx_toolkit). Genomes were assembled *de novo* using Tadpole (BBMap 35.49) (http://sourceforge.net/projects/bbmap) with *k*mer values generated by Kmergenie (9) and mapping to the NC_002593.1 reference (10) using BWA version 0.7.12 (11) and SAMtools version 1.2 (12). The *de novo* assembled contigs, BWA generated mapping, and trimmed reads were merged into a single FastQ file and then re-mapped to the NC_002593.1 reference using the Geneious R9 mapper (13) with low to medium sensitivity and 5 iterations. Gaps were filled manually from Sanger sequences. All four genomes showed a high degree of similarity, with 40.7% G+C content and sequence homology of 99.9%. Open reading frames (ORFs) were predicted using the Geneious R9 live annotation tool and compared to the NC_002593.1 reference with the larger of overlapping ORFs selected. One hundred eighteen ORFs were predicted for all four *Plxy*GV isolates, two fewer than the NC_002593.1 reference genome. The completed genomes are 100,980 bp (*Plxy*GV-C), 100,978 bp (*Plxy*GV-T), 101,004 bp (*Plxy*GV-K), and 100,980 bp (*Plxy*GV-M) in length.

Genomic differences arise in the position of ORF73 (which shares 58.6% nucleotide sequence identity with AcMNPV ORF91) and is truncated in PlxyGV-K. The regions of greatest sequence variation occur within two pairs of noncoding regions, which are almost identical in length (pair one, 2,596 bp and 2,516 bp; pair two, 1,340 bp and 1,414 bp) in the four isolates and NC_002593.1 reference genome. These are homologous repeat regions that are common features within baculovirus genomes (14).

### Nucleotide sequence accession numbers.

This whole-genome shotgun project has been deposited in DDBJ/EMBL/GenBank under the accession numbers KU529791 (*Plxy*GV-C), KU529792 (*Plxy*GV-K), KU529793 (*Plxy*GV-M), and KU529794 (*Plxy*GV-T). The versions described in this paper are the first versions, KU529791.1, KU529792.1, KU529793.1, and KU529794.1.
